# Profiling spermatogenic failure in adult testes bearing *Sox9*-deficient Sertoli cells identifies genes involved in feminization, inflammation and stress

**DOI:** 10.1186/1477-7827-8-154

**Published:** 2010-12-23

**Authors:** Aurélie Lardenois, Frédéric Chalmel, Francisco Barrionuevo, Philippe Demougin, Gerd Scherer, Michael Primig

**Affiliations:** 1 Inserm, U625, Université de Rennes 1, IFR140, Rennes, F-35042, France; 2 Institute of Human Genetics, University of Freiburg, Freiburg, D-79106, Germany; 3 Biozentrum, University of Basel, Basel, CH-4056, Switzerland; 4 Departamento de Genética e Instituto de Biotecnología, University of Granada, Granada, Spain

## Abstract

**Background:**

*Sox9 *(*Sry *box containing gene 9) is a DNA-binding transcription factor involved in chondrocyte development and sex determination. The protein's absence in testicular Sertoli nurse cells has been shown to disrupt testicular function in adults but little is known at the genome-wide level about molecular events concomitant with testicular break-down.

**Methods:**

To determine the genome-wide effect on mRNA concentrations triggered by the absence of *Sox9 *in Sertoli cells we analysed adult testicular tissue from wild-type versus mutant mice with high-density oligonucleotide microarrays and integrated the output of this experiment with regulatory motif predictions and protein-protein network data.

**Results:**

We report the genome-wide mRNA signature of adult testes lacking *Sox9 *in Sertoli cells before and after the onset of late spermatogenic failure as compared to fertile controls. The GeneChip data integrated with evolutionarily conserved *Sox9 *DNA binding motifs and regulatory network data identified genes involved in feminization, stress response and inflammation.

**Conclusions:**

Our results extend previous observations that genes required for female gonadogenesis are up-regulated in the absence of *Sox9 *in fetal Sertoli cells to the adult stage. Importantly, we identify gene networks involved in immunological processes and stress response which is reminiscent of a phenomenon occurring in a sub-group of infertile men. This suggests mice lacking *Sox9 *in their Sertoli cells to be a potentially useful model for adult human testicular failure.

## Background

Sex differentiation of mammalian males is controlled by the Y-chromosomal locus *Sry *(Sex-determining region of Y-chromosome) which, in cooperation with Steroidogenic factor 1 (*Sf1*), directly activates the expression of *Sox9 *[1]; for review, see [2]. This protein is an important transcription factor involved in various developmental processes and pathologies [3]. *Sox9 *is expressed in supporting cell precursors destined to become Sertoli cells which are required for normal testis morphology and germ cell development [4-6]; reviewed in [7,8]. *Sox9 *is actively imported into the nucleus during male gonad development [9,10] and specifically interacts with the ^A^/_T_ACAA^T^/_A _motif via its high mobility group (HMG) DNA binding and DNA bending domain [2,11]. The protein is thought to stimulate its own expression via a C-terminal transactivation domain together with *Sf1 *after the sex determination stage when *Sry *expression is repressed; reviewed in [2,12].

Human SOX9 was found to be mutated in patients suffering from campomelic dysplasia, a condition linked with abnormal skeletal development and, critically, perturbed male gonadogenesis or complete sex reversal [13,14]. This is in keeping with an important role for the rodent *Sox9 *protein in murine male gonad development initially suggested on the basis of its expression pattern [5,6]. Experiments addressing *Sox9*'s reproductive function were complicated by the fact that homozygous constitutive mutant mouse embryos die before the onset of sex differentiation [15]. Therefore, earlier work demonstrating the protein's critical role in the process of pre-natal testis development was based on Sertoli cell-specific partial or complete ablation of *Sox9 *in the mouse embryo prior to the initial stage of testis development [16,17]. Recently it was reported that Sertoli-cell specific deletion of *Sox9 *at embryonic day 14 (E14.0) - two and a half days after the sex determination stage - had no effect on the formation of seminiferous tubules and early adult testicular function; however, at late stages testicular architecture and spermatogenesis were disrupted [18]. These results suggest critical roles for *Sox9 *during pre-natal sex determination and in adult maintenance of spermatogenesis. However, nothing is known about the global impact of *Sox9*'s absence in adult Sertoli cells at the molecular level [19].

Here we investigate the testicular genome-wide mRNA concentration profile in adult mice bearing an AMH-Cre transgene and two *Sox9 *alleles with Cre recognition sites (AMH-Cre *Sox9*^flox/flox^). Testicular samples from mice whose Sertoli-cells lack *Sox9 *from E14.0 onwards were isolated before the onset of infertility at the age of 90 days post partum (dpp) and at 165 dpp, when the phenotype was clearly detectable. These samples were compared to controls homozygous for a functional *Sox9 *allele (*Sox9*^flox/flox^) taken at the same age. The output of the expression profiling study was integrated with a genome-wide search for evolutionarily conserved *Sox9 *binding sites to help identify potential direct targets and with regulatory network data to explore systemic effects in the mutant. Our results reveal a complex mRNA signature in part due to changing testicular cell proportions and to the up-regulation of genes involved in female gonad development but also possibly to the de-regulation of mouse genes involved in stress response and inflammation not previously reported in the context of spermatogenic failure.

## Methods

### Mouse strains

Mice bearing *Sox9*^flox/flox ^[20] and AMH-Cre transgenes [21] were crossed to obtain AMH-Cre *Sox9*^flox/flox ^mice which were genotyped as published [18].

### Testicular sample preparation

Total testis samples were prepared from C57BL/6 *Sox9*^flox/flox ^and AMH-Cre *Sox9*^flox/flox ^mutant animals at 3 (90 dpp) and 5.5 (165 dpp) month after birth. Testes of at least three animals for each time points were decapsulated and combined into duplicate pools using standard laboratory practice.

### Target synthesis, GeneChip hybridization and raw data production

Total RNA preparation, cRNA target synthesis and raw data production using Mouse Genome 430 2.0 GeneChips (Affymetrix) were essentially done as previously published [22].

### Expression data analysis

The microarray data were pre-processed and analyzed using the AMEN (Annotation, Mapping, Expression and Network analysis) suite of tools [23]. The data quality was verified by plotting the surface intensity distribution, 3'-5' RNA degradation and log2 signal distribution across samples. Data were normalized using the Robust Multi-Array Average (RMA) method as previously published and the global signal intensities from *Sox9*^flox/flox ^and mutant AMH-Cre *Sox9*^flox/flox ^samples were visualized using a Distance Matrix in combination with a dendrogram as published [22,24].

### Statistical filtration and classification

Probe sets yielding a signal higher than the detection threshold (median of the normalized dataset, cutoff 5.3) and a fold-change ≥2.0 between *Sox9*^flox/flox ^and AMH-Cre *Sox*9^flox/flox ^at 90 and 165 dpp were selected. A LIMMA statistical test (F-value adjusted with the False Discovery Rate method: p ≤ 0.01) was employed to identify significantly differentially expressed probe sets which were subsequently classified into two groups using the k-means algorithm (k = 2).

### Gene Ontology (GO) and transcription factor binding site (TFBS) enrichment

Enrichment of GO terms and predicted TFBSs were estimated with the Fisher exact probability using a Gaussian Hypergeometric test as previously published [22]. A GO term or a TFBS matrix was considered to be significantly enriched in a group of genes when the FDR-corrected *p*-value was ≤0.01 and the number of genes bearing this annotation or a TFBS was ≥5.

### Regulatory network analysis

The network representation was drawn using AMEN [23]. The protein-gene regulation data were downloaded from the TRANSFAC Professional Database release 2010.1 [25] and from Transcription Factor Encyclopedia (TFe, accessed May 1^st^, 2010).

### Prediction of TFBSs conserved across species

Transcriptional Start Sites (TSS) extracted from all_mrna.txt and refseqAli.txt UCSC mapping files were localized by associating annotated mouse protein-coding genes (mm9 genome) with their corresponding transcripts as defined in the gene2accession file provided by the NCBI [26]. TFBSs matrices from the TRANSFAC Professional Database release 2010.1 were predicted using the MATCH software [27] with the minSUM_good profile to minimize false negative and false positive predictions. Motif predictions were limited to a region of 1 kb upstream of the Transcriptional Start Site (TSS). Motifs displaying a core similarity score (CSS) and a matrix similarity score (MSS) of ≥0.8 were selected. To further reduce the number of false positives potential motifs had to be conserved [28]. For each prediction a cross-species conservation score was computed by averaging the base-by-base phastCons scores calculated between 30 vertebrates as provided by the UCSC genome browser [29]. Predicted motifs with a conservation score ≥0.8 were selected. Mouse genes whose promoters contained at least one predicted motif were used to calculate its enrichment as compared to the promoters of all annotated genes.

### MIAME Compliance

Raw data CEL files corresponding to 8 total testicular samples collected in duplicate at the age of 90 and 165 dpp from C57BL/6 *Sox9*^flox/flox ^and AMH-Cre *Sox9*^flox/flox ^mice, respectively, are available via the EBI's ArrayExpress under the accession number E-TABM-528 [30]. Normalized data are available for viewing at GermOnline [31].

## Results

### Experimental design and quality control

It was found earlier that Sertoli-cell specific deletion of *Sox9 *from E14 onwards had no effect on the fertility of young adults but caused late spermatogenic failure due to progressive degeneration of the seminiferous tubules [18]. To gain insight into which *Sox9*-dependent processes may contribute to this phenotype we compared the testicular mRNA profiles of phenotypically normal *Sox9*^flox/flox ^mice and mutant AMH-Cre *Sox9*^flox/flox ^mice. As a control, samples from both backgrounds were first taken at 90 dpp prior to the onset of the phenotype where testes from *Sox9*^flox/flox ^and AMH-Cre *Sox9*^flox/flox ^mice are morphologically and histologically indistinguishable. A second set of samples was prepared at 165 dpp which is approximately two weeks after the point where pathological changes first become histologically apparent, but well before testicular architecture breaks down completely in the AMH-Cre *Sox9*^flox/flox ^background [18]. Affymetrix Mouse Genome 430 2.0 high-density oligonucleotide GeneChips are extremely robust tools that yield highly reproducible data [32,33] and complex mammalian samples are amenable to reliable analysis when examined in duplicate [22,24,34]. We therefore used two independent pooled samples of 90 dpp and 165 dpp testes from *Sox9*^flox/flox ^and AMH-Cre *Sox9*^flox/flox ^mice to prepare total- and cRNA of uniformly high quality (Additional file 1 Figure S1A and B). GeneChip hybridization patterns were normal in all cases (Additional file 1 Figure S1C) and the signals displayed the expected RNA degradation and intensity distribution profiles (Additional file 1 Figure S1D and E).

### Testes from *Sox9*^flox/flox ^and AMH-Cre *Sox9*^flox/flox ^mice show distinct mRNA concentration profiles

**Figure 1:**
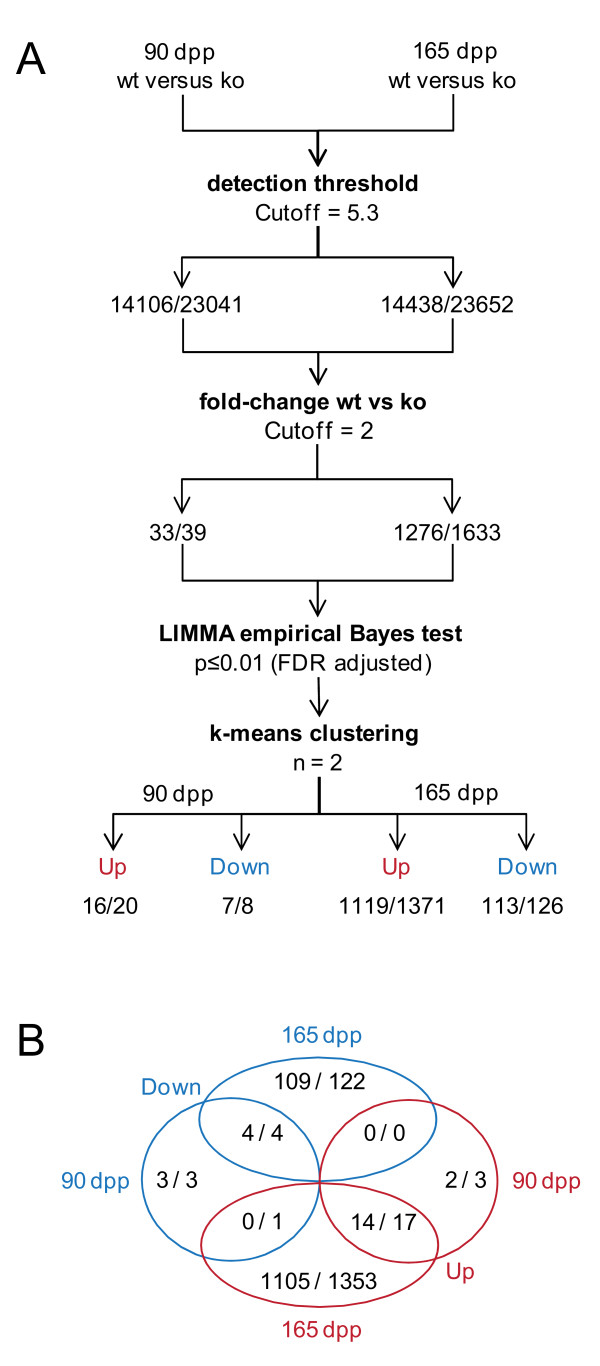
**Gene filtration strategy and output**. (A) A schematic drawing outlines the method used to identify and cluster genes showing statistically significant signal changes across the 90 and 165 dpp time points. The numbers of genes and probe sets are given. (B) A Venn diagram summarizes the distribution of genes into up and down categories at the time points given.

**Figure 2:**
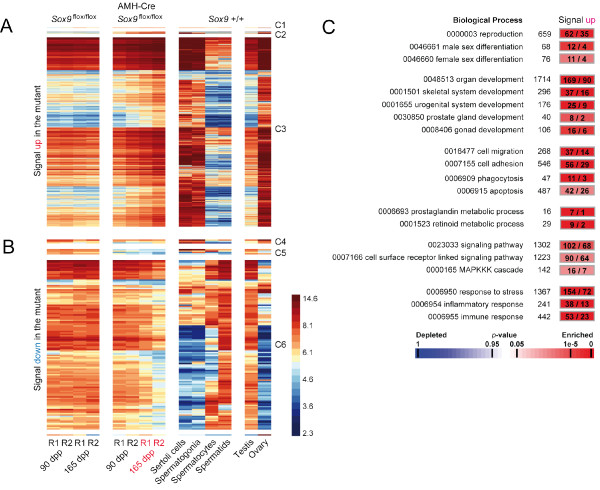
**Gene clustering and GO term analysis**. (A) A false-color heatmap shows cases of increasing (up) mRNA signal intensities across the replicates (R1, R2) for different total testis samples (top) at the time points given (bottom). They are compared to four different purified testicular cell types and two external controls (Testis, Ovary). Genes are grouped into three different clusters according to their signal patterns at different time points (C1-C3). A color scale for log2 transformed data is shown. (B) A similar display is given for genes that display weaker signals in the mutant samples (down), they were also grouped into clusters (C4-C5). (C) For genes showing stronger signals in the mutant (up) significantly enriched GO terms and their identification numbers are given (left) together with the number of genes associated with the term (middle) and the number of genes observed versus expected. A color scale for enriched and depleted terms is show at the bottom.

We first identified 14,106 and 14,438 genes, respectively, for which signals above the threshold level of detection (5.3 log2 units corresponding to the median intensity) were obtained at 90 dpp and 165 dpp (Additional File 2 Table S1). We next selected genes significantly differentially expressed (2-fold change, LIMMA test with FDR adjusted *p*-value < 0.01) and used the k-means clustering algorithm (n = 2) to group them into 16 genes showing stronger signals and 7 genes showing weaker signals in the mutant samples at 90 dpp (Figure 1A). The extremely low number of gene for which variations were found is in keeping with the lack of any detectable phenotype at the 90 dpp control time point as compared to the *Sox9*^flox/flox ^control. We then repeated the procedure with the 165 dpp samples and identified 1119 genes for which we measured increased signals and 113 genes associated with decreased signals (Figure 2A). This result is consistent with the morphological changes observed in testicular tissue after the onset of dysgenesis at the age of 5 months [18].

Among the loci for which signals are stronger in the mutant, only two were found exclusively at 90 dpp, 14 were observed in both samples and 1105 were detected only at 165 dpp. Similarly, three, four and 109 genes were found to be weaker only at 90 dpp, in both samples or only at 165 dpp, respectively (Figure 1B and Additional File 2 Table S1). These results show, as expected, that a substantial effect on RNA concentrations is detectable only at 165 dpp at the onset of testicular failure.

### Increasing and decreasing mRNA concentrations in AMH-Cre *Sox9*^flox/flox ^samples are associated with different testicular cell types and biological processes

We integrated the expression signals obtained with total testis samples from *Sox9*^flox/flox ^and AMH-Cre *Sox9*^flox/flox ^mice with our data from enriched wild-type Sertoli cells, spermatogonia, spermatocytes, spermatids and total testis samples [22] as well as ovary samples obtained from the GEO public array data repository (Methods) [35]. We note that the experimental design allows for determining overall mRNA concentration changes between replicate samples which may or may not be due to transcriptional effects. We refer to statistically significant changes in expression values between wild-type and mutant testes alternatively as stronger (increasing) and weaker (decreasing) signals which implies that more or less mRNA, respectively, is present in the given sample.

Figure 2A shows a heatmap summarizing expression signals for two genes showing stronger signals only at 90 dpp while no increase was found at 165 dpp (Class 1; *Fam181b*, *Igf1r*), 14 genes showing stronger signals at both time points (Class 2; *Aqp5*, *C3*, *Ccdc80*, *Ildr2*, *Hoxd10*, *Lrg1*, *Serpina3n*, *Spon1*, *Sult1e1*, *Thrsp*, *Timp1*, *Tnfrsf12a*, *Tspan8*, *Wwtr1*) and 1105 loci which pass our selection criteria (>2-fold) for a significant signal increase only at 165 dpp (Class 3; see Additional File 2 Table S1 which contains *find *and *filtering *functions to call up the complete expression data set for individual genes or groups of loci; see also GermOnline for expression data freely available for all genes represented on the Mouse Genome 430 2.0 GeneChip; [36]). For genes in Class 3 we observe the strongest signals in purified somatic wild-type Sertoli cells and spermatogonia as well as ovary (Figure 2A).

Figure 2B depicts the signals for three genes showing weaker signals only at 90 dpp (Class 4; *Kctd14*, *Schip1*, *LOC100044139*), four cases where signals were decreased in both samples (Class 5; *Clic6*, *Diras2*, *Sox6*, *Sox9*) and 109 loci whose transcript concentrations decrease in the mutant at 165 dpp (Class 6; Additional File 2 Table S1). As opposed to the genes shown in panel A we typically find the loci to display the strongest signals in purified pachytene spermatocytes and round spermatids; these results are confirmed by strong signals in testes and mostly weak ones in ovary (Figure 2B).

We next explored the biological processes associated with groups of genes showing signal changes among the sample set by determining if these groups were statistically significantly enriched for genes bearing specific Gene Ontology (GO) terms (Methods) (Figure 2C). While no GO terms were found to be enriched in the group showing decreased signals, we identified several relevant processes among the genes for which stronger signals are observed in the mutant. First, as anticipated, we found *Reproduction *(GO:0000003, 62 observed/35 expected by chance, *p*-value: 3.6 × 10^-5^), *Male sex differentiation *(0046661, 12/4, 4.5 × 10^-4^), and *Female sex differentiation *(0046660, 11/4, 3.8 × 10^-3^). A large group of genes was annotated as being involved in *Organ development *(0048513, 169/90, 1.3 × 10^-15^); consistently, we find enrichment of terms relevant for bone formation and the reproductive tract such as *Skeletal system development *(0001501, 37/16, 4.8 × 10^-6^) and development of the *Urogenital system *(0001655, 25/9, 2.3 × 10^-5^), *Prostate gland *(0030850, 8/2, 1.5 × 10^-3^), and *Gonad *(0008406, 16/6, 3.7 × 10^-4^).

At the cellular level we found two terms particularly pertinent for Sertoli cell- germ cell interactions as spermatogenesis progresses: *Cell migration *(0016477, 37/14, 3.9 × 10^-7^), and *Cell adhesion *(0007155, 56/29, 8 × 10^-6^) while *Phagocytosis *(0006909, 11/3, 5 × 10^-5^) and *Apoptosis *(0006915, 42/26, 3.9 × 10^-3^) reveal extensive absorption of cellular material and programmed cell death typical for Sertoli cells and meiotic germ cells, respectively. As far as biochemical processes important for reproduction are concerned we identified metabolism of *Prostaglandin *(0006693, 7/1, 7.6 × 10^-6^), and *Retinoid *(0001523, 9/2, 1.7 × 10^-5^).

Interestingly, the group of genes showing increased signals includes some involved in *Signaling *(0023033, 102/68, 1.6 × 10^-4^) and notably *Cell surface receptor linked signaling *(0007166, 90/64, 2.9 × 10^-3^) such as the *MAPKKK cascade *(0000165, 16/7, 7.5 × 10^-3^). These signal transduction pathways play roles in similarly enriched processes such as *Response to stress *(0006950, 154/72, 1.6 × 10^-19^) and *Inflammatory*-, (0006954, 38/13, 6.2 × 10^-2^) as well as *Immune response *(0006955, 53/23, 1.1 × 10^-7^).

These results indicate that reproducible mRNA concentration changes occur in AMH-Cre *Sox9*^flox/flox ^samples at the onset of testicular breakdown, and that the group of genes for which we find a signal increase contains more genes involved in male reproductive functions, female sex differentiation, stress response and inflammatory processes than would be expected to occur by chance. Lists of genes bearing specific GO annotations as described are available via Additional File 2 Table S1.

### Conserved regulatory motifs are enriched within the promoters of genes showing signal changes in *Sox9*^flox/flox ^versus AMH-Cre *Sox9*^flox/flox ^testes

**Figure 3:**
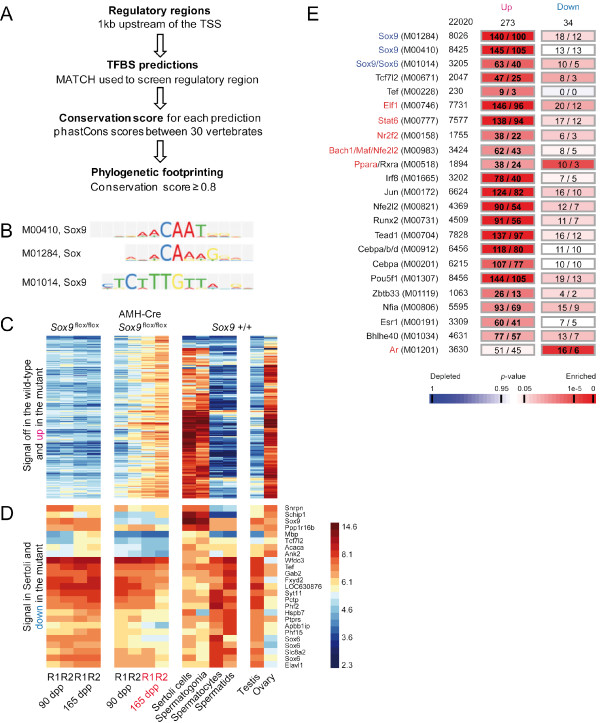
**Combined expression and motif prediction analysis**. (A) The method used to identify conserved transcription factor binding sites (TFBSs) is shown. (B) The identification numbers and the logos of three different *Sox9 *binding site matrices from TRANSFAC are given. (C) A heatmap is shown for genes for which mRNAs are detected only in mutant testicular samples (up) in comparison to purified testicular cells and external controls as in Figure 3. (D) A heatmap for genes showing weaker signals in the mutant (down) and above-background signals in purified Sertoli cells. The proximal promoters of the genes shown in panels C and D contain at least one predicted *Sox9 *binding site. A log2 scale is given. (E) Transcription factor symbols and their corresponding binding matrix identifiers (left), the total number of promoter sequences that contain them (middle), and the numbers of promoters observed versus expected by chance for genes falling into *increase *and *decrease *groups shown in panel A (right) are given. The total numbers in each category is given at the top of the columns. A color scale showing depletion and enrichment is shown at the bottom. Regulators for which we detect stronger or weaker signals in the mutant are given in red and blue, respectively.

To gain further insight into the regulatory processes that contribute to the RNA signature triggered by the absence of *Sox9 *in Sertoli cells, we first identified 202 genes for which mRNA was detectable only in mutant testes but not the normal controls. We found that the promoters of these genes - for which we find strong signals in purified Sertoli cells, spermatogonia and ovary but not in testis - were statistically significantly enriched for conserved target sites of *Sox9 *(Figure 3A-C, Methods). Moreover, we identified the motifs of regulators known or thought to be involved in Wnt signaling (*Tcf7l2*, *Zbtb33*/*Kaiso*), embryonic and post-natal development (*Maf*, *Nfia*, *Pou5f1*, *Runx2*, *Tead1*, *Tef*), steroid hormone signaling (*Esr1*, *Nr2f2*), stress response (*Nfe2l2*, *Ppara*) and immune function (*Bhlhe40*, *Cebpa*, *Cebpb*, *Elf1*, *Irf8*, *Jun*, *Stat6*) (Figure 3E).

We next selected 23 genes for which mRNA was detected in Sertoli cells and that showed weaker signals in AMH-Cre *Sox9*^flox/flox ^samples to determine if they were potential direct *Sox9 *targets. The majority of them showed the strongest signals in purified meiotic and post-meiotic germ-cells and among those for which the highest signals were observed in purified Sertoli cells only five (*Mpb*, *Tcf7l2*, *Acaca*, *Ank2 *and *Apbb1ip*) were below the threshold level of detection in the mutant and had at least one *Sox9 *binding site in their upstream promoter regions (Figure 3D). The only enriched motif identified in the group showing decreased signals in the mutant was the target site bound by the Androgen Receptor (*Ar*) which is involved in testosterone signaling (Figure 3E).

### Testicular failure in AMH-Cre *Sox9*^flox/flox ^mice is concomitant with extensive inflammatory and stress-response network activation

**Figure 4:**
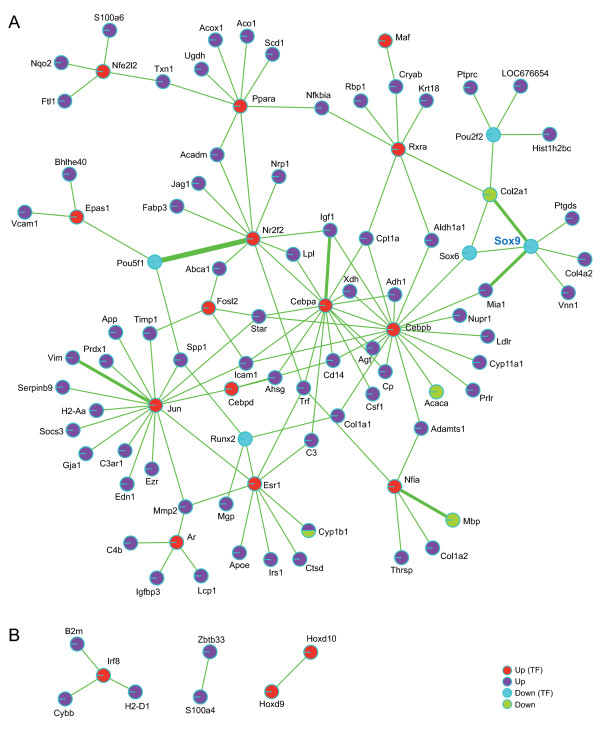
**Regulatory network analysis**. (A) The network is color coded with nodes given in red and blue for transcription factors (TF) showing stronger (up) or weaker (down) signals in the mutant, respectively. Target genes showing stronger or weaker signals in the mutant are given in dark blue (up) and green (down), respectively. The thickness of the edge represents the number of published protein/DNA interactions (see Methods). *Sox9 *is given in blue. (B) Three examples of small regulatory networks are given. A legend for the color code is shown.

To better understand the level of interconnectivity between transcription factors and target genes that responded to the *Sox9 *deletion we integrated the output of our analysis with published transcriptional regulatory data from TRANSFAC and TFe (Methods). We identified one large network including 14 transcription factors showing stronger signals in the AMH-Cre *Sox9*^flox/flox ^testes and five showing weaker ones (including *Sox9 *itself for which, as expected, no transcript was detected) (Figure 4A). Furthermore, we found a small complex (*Irf8 *and *B2m*/*Cybb*/*H2-D1 *targets) and two binary interactions (*Zbtb33*/*S100a4 *and *Hoxd9*/*Hoxd10*) (Figure 4B). The interactions typically reveal concordant patterns for transcription factors and their target genes in the group of genes showing increased signals in the mutant. Factors such as *Cebpb*, *Epas1*, *Irf8*, and *Jun *are involved in controlling immunological processes often via regulating cytokine gene expression. The network data also suggest a certain level of coordination between immune-, and stress response, development (*Cebpa*, *Fosl2*, *Hoxd9*, *Hoxd10*, *Maf*, *Nfe2l2*, *Nfia*, *Ppara*) and gene expression controlled by steroid hormone receptors (*Ar*, *Esr1*, *Nr2f2 *and *Rxra*).

In addition to *Sox9*, we identified four transcription factors which displayed weaker signals in AMH-Cre *Sox9*^flox/flox ^testes in spite of the fact that their target genes show a signal increase: *Sox6 *(development of the central nervous system, chondrogenesis and maintenance of cardiac and skeletal muscle cells), *Pou5fl *(embryonic stem cell pluripotency), *Pou2f2 *(B-cell maturation) and *Runx2 *(chondrocyte-, and osteoblast differentiation).

## Discussion

Targeted deletion of the *Sox9 *transcription factor in testicular Sertoli cells at E14.0, after the initial phase of sex determination, does not prevent spermatogenesis from being established but causes its progressive degeneration from the age of five months onwards. The phenotype includes morphological changes such as reduced testis size, loss of germ cells in approximately 30% of the tubules and an increased Leydig cell population [18]. We sought to better understand the molecular events underlying this effect by establishing whole-genome RNA concentration profiles of samples from mice bearing the *Sox9*^flox/flox ^allele as compared to AMH-Cre *Sox9*^flox/flox ^animals. To this end, we isolated material from both strains before (90 dpp) and after (165 dpp) the onset of testicular degeneration. Because of practical issues including small sample size and low frequency of transgenic off-spring, we analysed total testicular tissues rather than purified cell populations. In the analysis described here histological observations were combined with different types of expression-, motif prediction and gene regulation data to discern biological processes and to establish a genome-wide signature of a complex progressive male infertility phenotype. The raw data are available via the EBI's ArrayExpress repository (Methods) and normalized signals were integrated into Additional file 2 Table S1 and the GermOnline database.

*Sox9 *is typically (but not exclusively) involved in the transcriptional activation of its targets genes [19,37]. Therefore we first focused on the group of genes showing diminished mRNA concentrations in AMH-Cre *Sox9*^flox/flox ^testis. Among 126 probe-sets displaying a signal decrease in the AMH-Cre *Sox9*^flox/flox ^mutant samples we found 62 to show the strongest signals in purified meiotic and post-meiotic germ cells indicating an indirect effect since Sox9 was deleted only in Sertoli cells. Although the genes corresponding to 25 of them contained at least one match to a known *Sox9 *motif in their proximal promoter region almost none of them were reliably detected in purified Sertoli cells effectively ruling them out as direct candidates (with the caveat that the purification procedure may have affected their expression). Moreover, in many cases the signals are only weaker but not abolished. We conclude that for these genes we find decreased signals most likely because of diminishing germ cell populations in the tubules of mutant testis [18].

We reasoned that direct *Sox9 *Sertoli target genes should display decreased signals in 90 dpp and 165 dpp AMH-Cre *Sox9*^flox/flox ^mutant samples, at least one *Sox9 *binding motif should be present in their proximal promoter regions and their mRNA should be detectable in purified wild-type Sertoli cells. The loci we found using these three selection criteria include *Acaca *(acetyl-Coenzyme A carboxylase alpha) which is involved in fatty acid metabolism; its deletion causes an embryonic lethal phenotype [38], *Mpb *(Myelin basic protein) which encodes a component of the myelin sheath in the central nervous system as well as other splice variants associated with diverse cellular functions [39], and *Ank2 *(Ankyrin 2) which is known to be involved in brain and muscle development [40,41]. Furthermore, we identified *Sox6 *which is predominantly expressed in the male germline but for which mRNA is also detectable in Sertoli cells (GermOnline; [22,42]). It was demonstrated that Sox6 expression is lost in *Sox9*-deficient limb buds [43], that the human SOX6 promoter is bound and activated by *Sox9 *in chondrogenic cells [44] and that *Sox6 *helps *Sox9 *activate a target gene in cartilage [45]. It is possible that the weaker signal for *Sox6 *is due to diminished germ cell numbers at 165 dpp. However, we observe a two-fold signal reduction already at 90 dpp when the germ cell content of the seminiferous tubules is unaffected. It is therefore conceivable that *Sox6 *plays an unexpected role in Sertoli cells and that its expression in this cell type (but not in germ cells) requires *Sox9*. Another potentially interesting gene we identified is *Tcf7l2 *(transcription factor 7-like 2) which encodes a protein involved in Wnt signaling [46,47]. It is known that *Sox9 *inhibits Wnt signaling in chondrocytes [48] and that abnormal activation of the Wnt pathway in mouse Sertoli cells leads to degeneration of tubules and infertility [49,50], a phenotype reminiscent of the one reported for AMH-Cre *Sox*9^flox/flox ^mice [18]. We speculate that perturbed *Tcf7l2*-dependent Wnt signaling might contribute to late-onset infertility in the absence of *Sox9 *in Sertoli cells.

Genes that show higher mRNA concentrations in the mutant testis or that are detectable only in pathological tissue reflect increasing cell populations (Leydig cells) and, predominantly, cellular stress. Examples include the Leydig cell markers *Cyp17*, *Cyp11a1*, *Cyp21a1*, and *Star *[51-53] or *Nr2f2 *[54] as well as genes that are involved in cell adhesion (*Cml5*), structural components such as collagens (*Col4a5*, *Col11a1*), keratin (*Krt18*), metalloproteases (*Adam8*, *Adam10*, *Adamts1*, *Adamts5*) and an inhibitor of this type of enzyme (*Timp1*). We also identified *Wnt4 *which is involved in female sex determination [55-57] (for review see [58]); it was observed previously that the absence of *Sox9 *in Sertoli cells triggers de-repression of genes involved in this process during pre- and early post-natal stages [16,17]. It is tempting to speculate that the phenomenon, due to dedifferentiation of mature Sertoli cells, persists until late adulthood. This is in keeping with the recent observation that a deletion of *Foxl2 *(the antagonist of *Sox9 *in female gonads) in ovarian follicle cells causes granulosa and theca cells to acquire Sertoli-, and Leydig cell-like features such as testosterone production [59] (for review, see [60]).

A potentially interesting result of our study is the strong immunological component of the RNA signature. This phenomenon is clearly reflected by the gene networks of *Jun *and *Cebpb *in particular: most of their target genes which show increased or even specific signals in AMH-Cre *Sox9*^flox/flox ^testes (such as *C3ar1*, *Cd44*, *Cd14*, *Cebpa*, *Cxcl13*, *H2-Aa*, *Icam1*, *Ildr2*, *Ly6d*, *Maob*, *Nupr1*, and *Prdx1*) are involved in immunological or inflammatory processes (see Mouse Genome Database for references). Interestingly, we also observed loci that play roles in down-regulating the immune response which likely reflects a stress response mechanism activated in increasingly damaged testicular tissue: for example, we found two serine protease inhibitors *Serpinb6b *(Serine (or cysteine) peptidase inhibitor, clade B, member 6B) and *Serpina3n*, [61], which are known to act on Granzymes A and B proteases secreted by cytotoxic T-lymphocytes [62,63]. Cultured human Sertoli cells - which are perhaps in a similar state of stress as *Sox9*-negative Sertoli cells in degenerating testicular tissue - secrete *Serpina3n *which was found to bind and inhibit Granzyme B, revealing a direct mechanism of Sertoli cell-dependent immunoprotection [64]. We note that Serpins are also produced by Leydig cells [65] and their strong mRNA signals may therefore at least in part be due to the proportional increase of the Leydig cell population in AMH-Cre *Sox9*^flox/flox ^samples at 165 dpp [18]. In this context it is interesting that *Serpina5*/*Pci *plays an important role in male reproduction since homozygous *Serpina5*-/- mice cannot maintain the Sertoli cell-dependent blood-testis barrier and become infertile [66]. We found that *Serpina5 *showed an mRNA signal increase at 165 dpp (GermOnline, Additional File 1) which may in part reflect a compensatory mechanism stemming the disruption of seminiferous tubules. Moreover, we observed strongly elevated mRNA concentrations for *Spp1 *(Secreted phosphoprotein 1) a protein thought to play a protective role by inhibiting apoptosis and by modulating the immune response [67].

Finally, it is remarkable that some mouse genes that display increased mRNA concentrations in mutant male gonads have human orthologs that appear to be up-regulated in the testes of different types of infertile patients showing various degrees of spermatogenic failure [68]. This group of loci includes *Adamts5 *(a disintegrin-like metallopeptidase with thrombospondin type 1 motif, 5; involved in osteoarthritis, [69]), *Clec2b *(C-type lectin domain family 1 involved in platelet activation and aggregation, [70]), *Ctsc *(Cathepsin C required for the activation of Granzymes A and B, [71]), *Ldlr *(Low density lipoprotein receptor involved in cholesterol homeostasis, induced by inflammation, [72]), and *Samd9l *(Sterile alpha motif domain containing 9-like involved in cytokine signaling [73]). We note that these genes are mostly involved in immunological phenomena.

## Conclusions

This study aimed at establishing a genome-wide mRNA signature associated with Sertoli cell-specific deletion of the *Sox9 *transcription factor to better understand the molecular events that are concomitant with - but not causative of - testicular failure. Our results suggest that *Sox9 *is required in Sertoli cells to prevent sex reversal until late adulthood and that AMH-Cre *Sox9*^flox/flox ^mice are a potentially useful model system to study testicular dysgenesis syndromes [74] and inflammatory processes that might ultimately contribute to the formation of malignant tumors [68]. The data pave the way for more detailed histological examinations of both the *Sox9 *mutant mouse model and patients presenting with progressive testicular failure to confirm at the protein level what our microarray study has revealed at the mRNA level.

## Competing interests

The authors declare that they have no competing interests.

## Authors' contributions

AL and FC analysed and interpreted expression, promoter motif prediction and regulatory network data, FB prepared the testicular samples, PD produced and quality controlled raw microarray data, GS designed research, MP interpreted data and wrote the manuscript. All authors read and approved the manuscript.

## Supplementary Material

Additional file 1 **Supplemental Figure S1: RNA and data quality controls**. (A) Total RNAs are shown from phenotypically normal (*Sox9*^flox/flox^) and mutant (AMH-Cre *Sox9*^flox/flox^) replicate samples as indicated. The molecular weight standard (MW) is given. (B) cRNA samples which were in some cases pooled together are summarized. (C) False-color images of the GeneChips hybridized with replicate targets (R1, R2) prepared from normal and mutant samples at the time-points indicated are shown to control for hybridization artifacts. (D) A plot of probe intensities (y-axis) against oligonucleotide probes (x-axis) is shown. (E) A box plot displays log2 intensities (y-axis) for all samples (x-axis) before normalization. Click here for file

Additional file 2 **Supplemental Table S1: Gene annotation and expression profiling output**. This file is in the MS xls format and contains information about genes including identifiers, probe set IDs, GO terms and color-coded, normalized, and log2 transformed expression data. Users are able to call up data for individual genes or lists of loci using the search and filtering options of MS Excel. Click here for file
