# Supplementary figures and images for: Profiling spermatogenic failure in adult testes bearing Sox9-deficient Sertoli cells identifies genes involved in feminization, inflammation and stress
Source: Reprod Biol Endocrinol. 2010 Dec 23;8:154. doi: 10.1186/1477-7827-8-154 (PMC3024295; doi:10.1186/1477-7827-8-154)

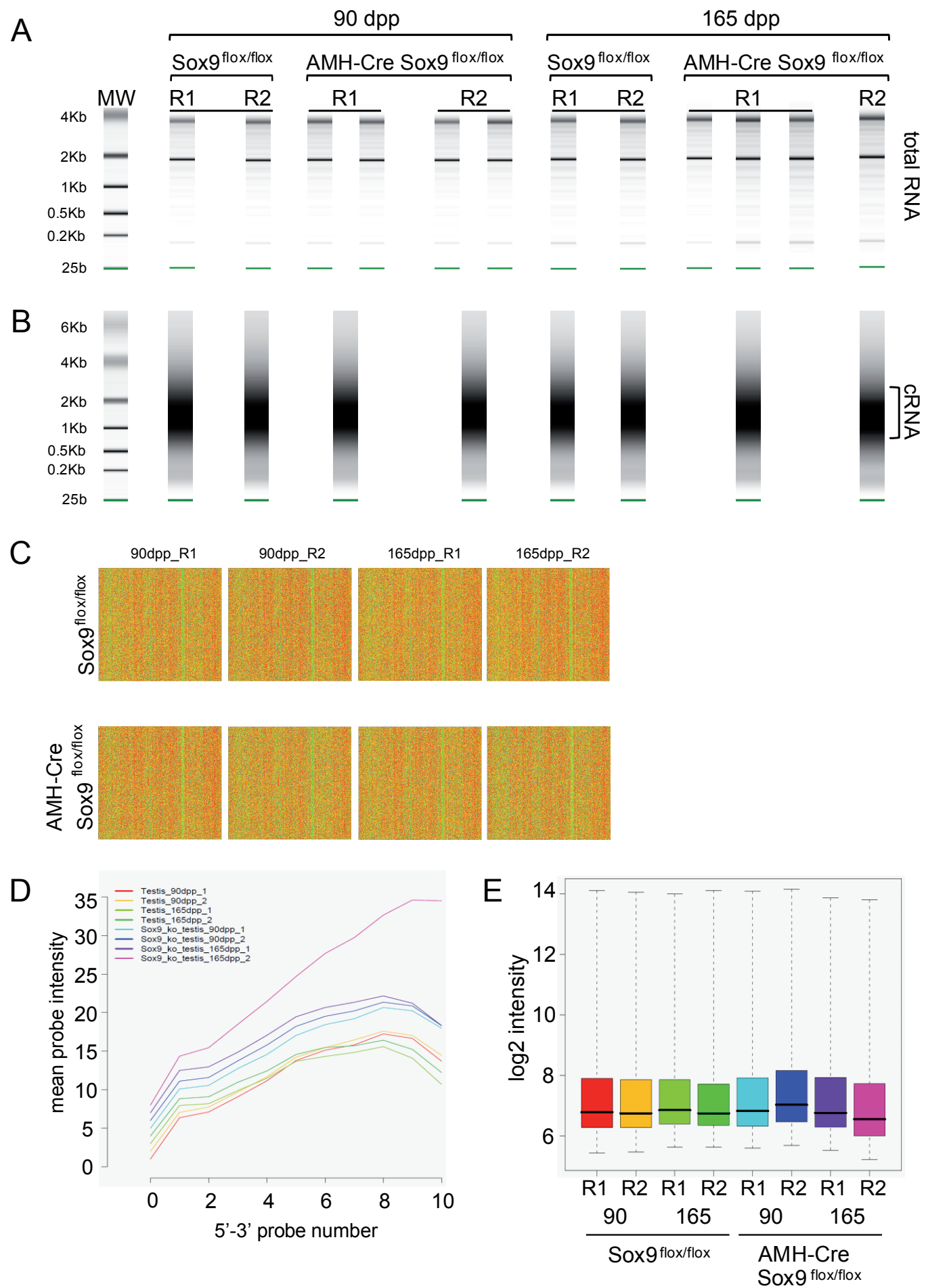

Supplement: Additional file 1 — Supplemental Figure S1: RNA and data quality controls. (A) Total RNAs are shown from phenotypically normal (Sox9flox/flox) and mutant (AMH-Cre Sox9flox/flox) replicate samples as indicated. The molecular weight standard (MW) is given. (B) cRNA samples which were in some cases pooled together are summarized. (C) False-color images of the GeneChips hybridized with replicate targets (R1, R2) prepared from normal and mutant samples at the time-points indicated are shown to control for hybridization artifacts. (D) A plot of probe intensities (y-axis) against oligonucleotide probes (x-axis) is shown. (E) A box plot displays log2 intensities (y-axis) for all samples (x-axis) before normalization. [file 1477-7827-8-154-S1.PDF]
